# Association between Acute Care Accessibility and in-Hospital Mortality among Patients with Acute Ischemic Stroke

**DOI:** 10.31662/jmaj.2024-0249

**Published:** 2025-02-21

**Authors:** Yusuke Sasahara, Yasufumi Gon, Eisuke Hida

**Affiliations:** 1 Department of Biostatistics and Data Science, Osaka University Graduate School of Medicine, Suita, Japan; 2 Department of Medical and Health Information Management, National Cerebral and Cardiovascular Center, Suita, Japan; 3 Department of Neurology, Osaka University Graduate School of Medicine, Suita, Japan; 4 Academic Clinical Research Center, Osaka University Hospital, Suita, Japan

**Keywords:** acute ischemic stroke, acute care accessibility, acute care density index, home-to-hospital distance, in-hospital mortality

## Abstract

**Introduction:**

Acute ischemic stroke (AIS) can lead to sequelae or death if not treated promptly. Patients residing in areas with limited acute care access may not receive prompt treatment; however, the association between accessibility to acute care and discharge outcomes in patients with AIS remains controversial. This study aimed to clarify the association between acute care density index (ACDI) and home-to-hospital distance and in-hospital mortality in patients with AIS.

**Methods:**

Using the Japanese registry of all cardiac and vascular diseases-diagnosis procedure combination database, we examined 525,689 patients (from April 2015 to March 2020). Hospital accessibility was assessed using ACDI and home-to-hospital distance. The patient residences were classified as urban, rural, or depopulated.

**Results:**

In urban areas, ACDI was associated with in-hospital mortality, with adjusted odds ratios for Q2, Q3, and Q4 compared with Q1 of 1.16 (95% confidence interval: 1.02-1.31), 1.23 (1.10-1.39), and 1.35 (1.19-1.53), respectively. Similar trends were observed in rural areas. In depopulated areas, home-to-hospital distance exceeding the median was associated with a reduction in in-hospital mortality, with adjusted odds ratios for Q3 and Q4 compared with Q1 of 0.84 (0.74-0.95) and 0.78 (0.68-0.89), respectively.

**Conclusions:**

A lower ACDI was associated with higher in-hospital mortality in both urban and rural areas, whereas longer home-to-hospital distance was not necessarily associated with higher in-hospital mortality.

## Introduction

Acute ischemic stroke (AIS) is a serious condition that can lead to sequelae or death if not treated promptly ^(1)^. Patients residing in areas with limited access to acute care, such as depopulated regions, may face challenges in receiving prompt treatment ^(2), (3)^. However, the association between accessibility to acute care and discharge outcomes in patients with AIS remains controversial ^(4)^.

To measure accessibility to acute care, both the supply-demand balance for medical services and transport distances must be considered ^(5)^. The Japan Medical Association Research Institute (JMARI) has been providing indicators for the density of acute care (acute care density index [ACDI]) since 2014 ^(6)^; it was designed to identify areas with excessive or inadequate volumes of acute care and has served as basic data for the development of regional medical plans ^(7), (8)^. Furthermore, ACDI has been used to visualize regional disparities in acute care resource allocation ^(7), (8), (9), (10), (11), (12), (13)^. However, the association between ACDI and in-hospital mortality in patients with AIS has not been assessed.

Although several studies have shown an association between longer home-to-hospital distance and increased mortality ^(4), (14), (15)^, their results have been inconclusive ^(16), (17), (18)^. Home-to-hospital distance tends to be shorter in cities and longer in the countryside, owing to which mortality is generally higher in the countryside than in cities ^(19), (20), (21), (22)^. When assessing the association between home-to-hospital distance and in-hospital mortality, stratifying the data by population size is necessary owing to differences in medical systems, living environments, regional lifestyles, and demographic heterogeneities. However, few studies have assessed this association on a nationwide scale stratified by population size in Japan. This study aimed to clarify the association between ACDI and home-to-hospital distance and in-hospital mortality in patients with AIS.

## Materials and Methods

### Diagnosis procedure combination (DPC) database

This retrospective, nationwide, observational cohort study used data from the Japanese registry of all cardiac and vascular diseases (JROAD)-DPC database. The JROAD study was launched in 2004 to assess the clinical practices of cardiovascular care in Japanese institutions; the DPC is based on data from the Japanese DPC/Per-Diem Payment System. The JROAD-DPC database is a combination of JROAD- and DPC-based claims data. Detailed methodologies for JROAD and JROAD-DPC have been published ^(23)^.

### Patients

**Figure 1.**
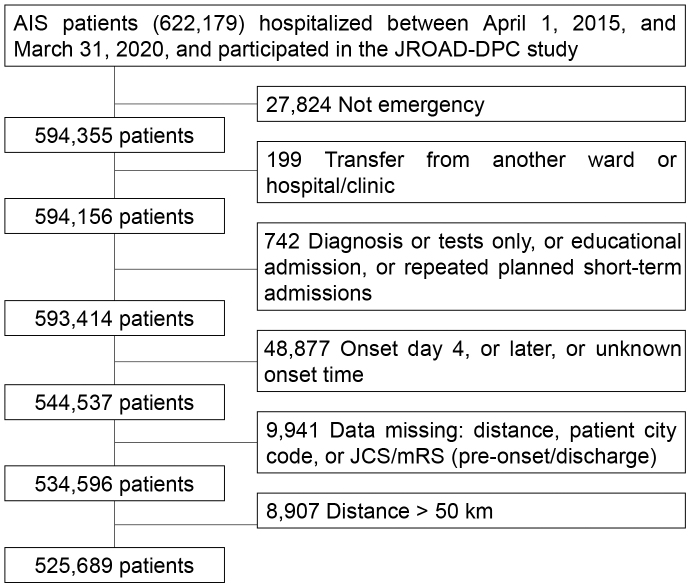
Flow chart of patient selection.

Cases were recruited considering the principal disease using the International Classification of Diseases, 10^th^ Edition code of I63 among those with a hospitalization period between April 1, 2015 and March 31, 2020 (Figure 1); patients younger than 18 years were excluded. This study also excluded the following cases: non-emergency hospitalizations; transfers from a different hospital; diagnostic tests only; educational hospitalizations; planned hospitalizations; >4 days after onset or with unknown onset time; missing data; unknown residence; unknown severity; and home-to-hospital distance exceeding 50 km ^(24)^ deemed to have occurred outside the daily living area.

### Hospital certification

Previous studies have demonstrated that admission to primary stroke centers (PSCs) influences in-hospital mortality ^(25)^. However, since PSC certification in Japan began in 2019, it was incomplete during our study period (2015-2020). Therefore, we referred to the Japan Stroke Society’s (JSS) official website ^(26)^ to identify hospitals certified as training and education hospitals; these certifications reflect the availability of specialized staff and equipment and can serve as robust indicators of hospital specialization. We assigned a certification flag to individual patient-level records and adjusted for this factor in the analysis.

### Secondary medical area classification, ACDI, and home-to-hospital distance

Patients residing in secondary medical areas were classified into three levels based on the 2015 JMARI definition ^(10), (27), (28)^: urban (regions with a population exceeding 1 million, or a population density exceeding 2,000 persons per square kilometer); rural (regions with a population exceeding 200,000, or over 100,000 and a population density exceeding 200 persons per square kilometer); and depopulated (regions that do not fall into either of the above categories).

ACDI quantifies the availability of acute medical care for managing residents and has been cited in JMARI working papers ^(7), (8), (9), (10), (11), (12), (13)^. This indicator was developed using two steps: (1) assessing the ability of each hospital to provide acute care based on the number of general anesthesia cases per year and bed volume and (2) distributing acute care capacity to regions according to travel time from the hospital and population size. A higher score indicated that the residents had easier access to acute care. If the index was 1.0, the ability of the area to provide acute care was at the national average level; if it was above 1.2, it was adequate; and if it was above 1.5, there was room to spare. Conversely, if it was ≤0.8, it was low, and if it was ≤0.6, it was quite low. The secondary medical area classification and ACDI data were obtained from the Secondary Medical Area Database Version 10.0.2 (Wellness Co., Ltd.) ^(29)^.

Home-to-hospital distance was defined as the distance between the coordinates of each postal code, which was determined using the GEODIST function in SAS version 9.4 (SAS Institute, Cary, NC, USA).

### Statistical analyses

Continuous and categorical variables were summarized as median (interquartile range) and percentage, respectively. Comparisons between groups were performed using the Kruskal-Wallis test and Pearson’s chi-square test. Interquartile trends were assessed using the Jonckheere-Terpstra (one-sided) and Cochran-Armitage (one-sided) trend tests. The prescription of a recombinant tissue plasminogen activator or endovascular treatment (receipt processing codes: 710010020, 710010021, 710010614, 710010640, 710010843, 710010846, 710010846, and 710011072) was defined as reperfusion therapy. Generalized linear mixed models, including hospitals as random effects, were used to assess the association between ACDI and home-to-hospital distance and in-hospital mortality. Fixed effects included ACDI, home-to-hospital distance, age, sex, hypertension, dyslipidemia, diabetes, congestive heart failure, atrial fibrillation, renal disease, obesity, smoking, modified Rankin scale score before onset, Japan Coma Scale (JCS) score at admission, emergency medical service (EMS), reperfusion therapy, cerebral rehabilitation, Japan Stroke Association-accredited training and education hospital admissions, and bed volume. All statistical analyses were performed using SAS. The nationwide distributions of ACDI, home-to-hospital distance, and in-hospital mortality were visualized using the heatmap function provided by the Secondary Medical Area Database ^(29)^.

### Ethics statement

The study was approved by the Research Ethics Review Board of the National Cerebral and Cardiovascular Center (R21041) and complied with the Declaration of Helsinki. The requirement for consent to provide individual information was waived based on the opt-out principle.

## Results

### Patient characteristics according to secondary medical area

From the JROAD-DPC database, data on 622,179 cases of AIS between April 2015 and March 2020 were extracted. The cases were filtered according to the inclusion and exclusion criteria, and 525,689 cases were included in the analysis (Figure 1). The baseline characteristics of the study population are presented in Table 1. The median ages were 76, 77, and 79 years for the urban, rural, and depopulated areas, respectively (presented hereafter in the order of urban, rural, and depopulated areas); as the ages increased, the population size decreased. The percentage of patients with severe JCS (JCS III) at admission also increased as the population size decreased (4.1%, 4.7%, and 5.0%, respectively). The percentage of admissions to the JSS training and teaching hospitals decreased as the population size decreased (83.6%, 83.4%, and 76.6%, respectively). The number of beds was highest in rural areas and lowest in depopulated hospitals (482 and 388, respectively). The median ACDI was highest in rural areas and lowest in depopulated areas (1.06 and 0.93, respectively). The median home-to-hospital distance increased as the population size decreased (2.95, 5.14, and 8.71 km, respectively).

**Table 1.** Patient Demographics, Hospital Admissions, and Residence.

	Secondary medical area				
	Overall	Urban	Rural	Depopulated	p-value
	n = 525,689	n = 209,843	n = 266,296	n = 49,550	
Age	77 (68, 84)	76 (68, 84)	77 (68, 85)	79 (70, 86)	<0.0001^*^
Male (%)	305,126 (58.0)	123,363 (58.7)	154,013 (57.8)	27,750 (56.0)	<0.0001^†^
Comorbidities					
Hypertension (%)	275,819 (52.4)	107,354 (51.1)	141,819 (53.2)	26,646 (53.7)	<0.0001^†^
Hyperlipidemia (%)	160,902 (30.6)	67,001 (31.9)	80,937 (30.3)	12,964 (26.1)	<0.0001^†^
Diabetes mellitus (%)	134,503 (25.5)	52,821 (25.1)	69,761 (26.2)	11,921 (24.0)	<0.0001^†^
Congestive heart failure (%)	46,433 (8.8)	16,842 (8.0)	24,274 (9.1)	5,317 (10.7)	<0.0001^†^
Atrial fibrillation (%)	115,134 (21.9)	43,523 (20.7)	59,863 (22.4)	11,748 (23.7)	<0.0001^†^
Renal disease (%)	23,781 (4.5)	9,705 (4.6)	12,085 (4.5)	1,991 (4.0)	<0.0001^†^
Obesity (%)	121,398 (23.0)	48,451 (23.0)	61,790 (23.2)	11,157 (22.5)	0.0039^†^
Smoking (%)	159,052 (34.3)	65,007 (35.6)	80,131 (33.8)	13,914 (30.9)	<0.0001^†^
Pre-onset modified Rankin Scale score (%)					
Grade 0 (%)	251,009 (47.7)	102,184 (48.7)	126,731 (47.5)	22,094 (44.5)	<0.0001^†^
Grade 1 (%)	95,399 (18.1)	37,389 (17.8)	47,675 (17.9)	10,335 (20.8)	<0.0001^†^
Grade 2 (%)	63,611 (12.1)	25,096 (11.9)	32,572 (12.2)	5,943 (11.9)	0.0126^†^
Grade 3 (%)	48,068 (9.1)	19,534 (9.3)	24,366 (9.1)	4,168 (8.4)	<0.0001^†^
Grade 4 (%)	47,521 (9.0)	18,426 (8.7)	24,435 (9.1)	4,660 (9.4)	<0.0001^†^
Grade 5 (%)	20,081 (3.8)	7,214 (3.4)	10,517 (3.9)	2,350 (4.7)	<0.0001^†^
JCS score upon admission					
0 (%)	252,878 (48.1)	101,296 (48.2)	128,616 (48.3)	22,966 (46.3)	<0.0001^†^
1-digit code (%)	201,247 (38.2)	81,166 (38.6)	100,583 (37.7)	19,498 (39.3)	<0.0001^†^
2-digit code (%)	47,680 (9.0)	18,572 (8.8)	24,510 (9.2)	4,598 (9.2)	<0.0001^†^
3-digit code (%)	23,765 (4.5)	8,767 (4.1)	12,516 (4.7)	2,482 (5.0)	<0.0001^†^
Traits of the admitting hospital					
Training and educational institution (%)	435,679 (82.8)	175,549 (83.6)	222,147 (83.4)	37,983 (76.6)	<0.0001^†^
Bed count	455 (327, 606)	451 (326, 613)	482 (340, 609)	388 (300, 460)	<0.0001^*^
Traits of the residential area					
ACDI	1.00 (0.77, 1.22)	0.99 (0.74, 1.10)	1.06 (0.84, 1.24)	0.93 (0.81, 1.24)	<0.0001^*^
Distance from the admitting hospital (km)	4.12 (2.14, 8.03)	2.95 (1.66, 5.00)	5.14 (2.63, 9.62)	8.71 (3.64, 17.06)	<0.0001^*^

Age, bed count, ACDI, and distance from the admitting hospital are presented as medians (first and third quartiles). Other items are presented as frequencies (%). ^*^Kruskal-Wallis test; ^†^Pearson’s chi-square test. ACDI, acute care density index; JCS, Japan Coma Scale.

### Nationwide ACDI, home-to-hospital distance, and in-hospital mortality distribution

**Figure 2.**
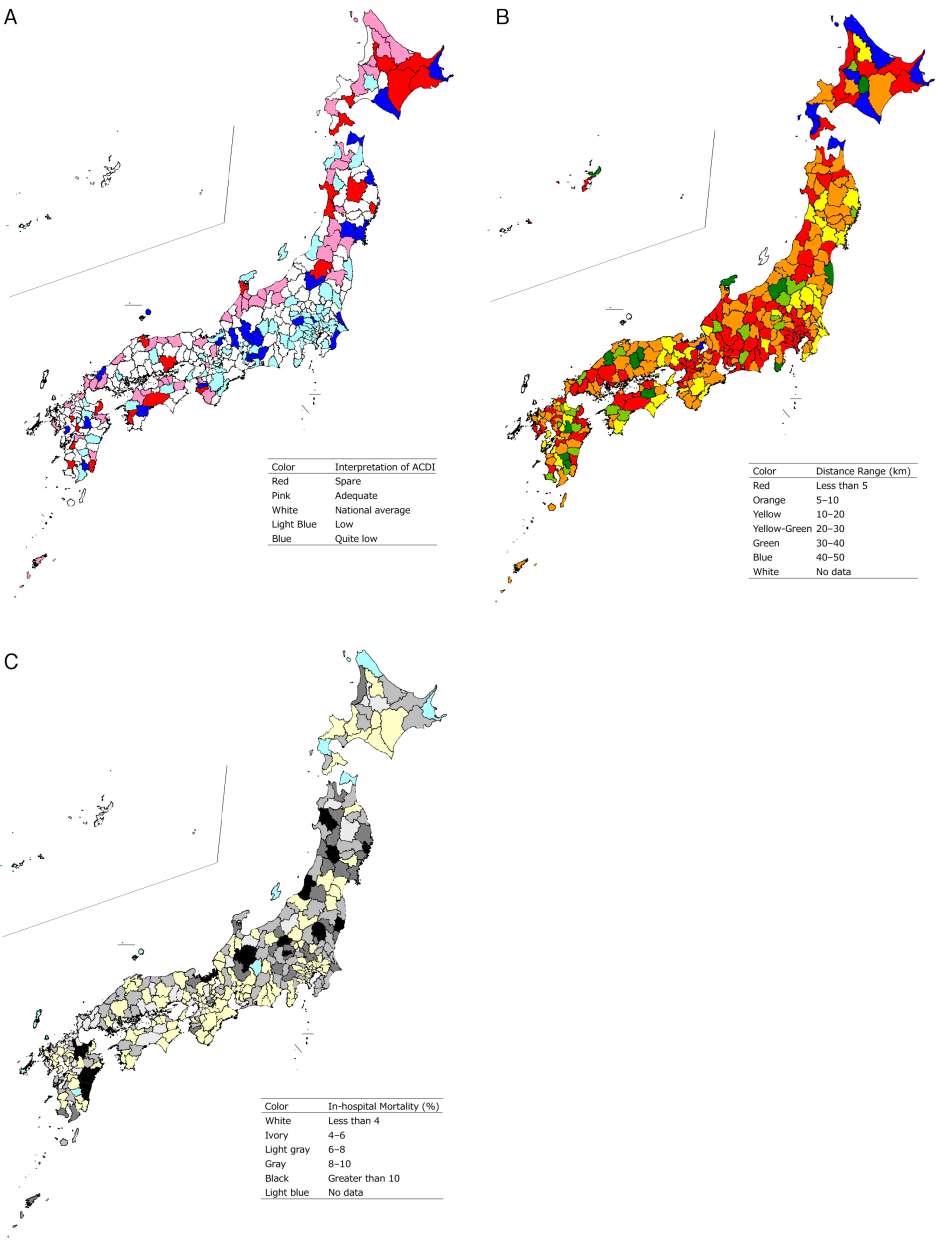
A. Nationwide ACDI Distribution. B. Nationwide Home-to-hospital Distance Distribution. C. Nationwide In-hospital Mortality Distribution.

**Figure 3.**
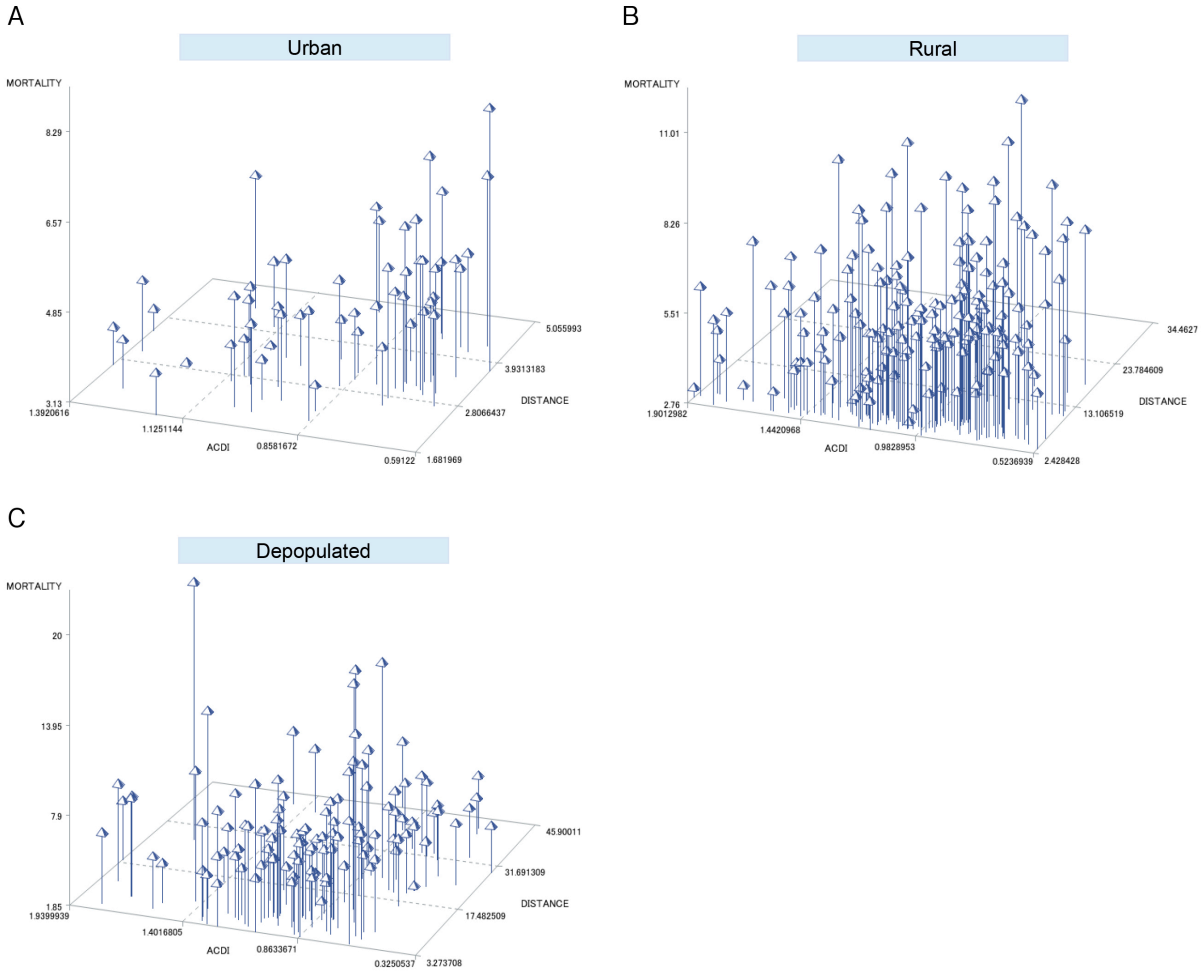
distribution of ACDI, Home-to-hospital Distance, and In-hospital Mortality

ACDI, home-to-hospital distance, and in-hospital mortality of the secondary medical areas where the patients resided were visualized using a heatmap (Figure 2). Regarding ACDI, blue (quite low ACDI) was observed nationwide in the countryside, whereas light blue (low ACDI) was concentrated in metropolitan areas such as Tokyo and Kanagawa. Red and pink (spare or adequate ACDI) were observed in areas such as Hokkaido and along the Sea of Japan coast, whereas they were rare in metropolitan areas (Figure 2A). Regarding home-to-hospital distance, blue and green (long distances) were common in the countryside, with distances exceeding 40 km in parts of Hokkaido, Aomori, and Shiga. In contrast, red (short distances) was prominent in metropolitan areas such as Tokyo and Osaka (Figure 2B). Regarding in-hospital mortality, regions with high mortality (exceeding 10%) were observed in Tohoku, northern Kanto, Hokuriku, and Kyushu, whereas they were not observed along the Pacific coast of Honshu or in Shikoku (Figure 2C). Three-dimensional scatter plots illustrating ACDI, home-to-hospital distance, and in-hospital mortality for each secondary medical area are shown in Figure 3. In-hospital mortality increased with a decrease in ACDI and an increase in home-to-hospital distance in urban areas; however, rural and depopulated areas did not exhibit a clear trend.

### Trends in severity, care, and in-hospital mortality

The trends in severity, care, and in-hospital mortality according to ACDI are presented in Table 2. Urban areas showed a trend toward decreasing reperfusion therapy and increasing in-hospital mortality as ACDI decreased. Rural areas showed an increasing trend in the proportion of severe cases (JCS 3-digit code) as ACDI decreased. The percentage of patients receiving reperfusion therapy admitted to hospitals accredited by the JSS decreased as ACDI decreased. In-hospital mortality increased as ACDI decreased. Depopulated areas showed a trend toward increasing reperfusion therapy as ACDI decreased.

**Table 2.** Trends in Severity, Care, and Outcomes According to the ACDI.

	ACDI					
	Overall	1st quartile	2nd quartile	3rd quartile	4th quartile	Trend test
						p-value
**Urban**						
ACDI	1.39-0.59	1.39-1.10	1.10-0.99	0.99-0.74	0.74-0.59	―
Number of patients	209,843	56,444	45,133	58,004	50,262	―
JCS score upon admission						
0 (%)	101,296 (48.2)	27,263 (48.3)	22,599 (50.0)	28,456 (49.0)	22,978 (45.7)	<0.0001^*^
1-digit code (%)	81,166 (38.6)	22,318 (39.5)	15,824 (35.0)	22,472 (38.7)	20,552 (40.9)	<0.0001^*^
2-digit code (%)	18,572 (8.8)	4,518 (8.0)	4,710 (10.4)	4,805 (8.2)	4,539 (9.0)	0.0134^*^
3-digit code (%)	8,767 (4.1)	2,333 (4.1)	1,994 (4.4)	2,263 (3.9)	2,177 (4.3)	0.4145^*^
EMS utilization (%)	128,701 (61.3)	33,759 (59.8)	28,079 (62.2)	36,129 (62.2)	30,734 (61.1)	<0.0001^*^
Reperfusion therapy (%)	22,565 (10.7)	6,456 (11.49)	4,798 (10.6)	5,695 (9.8)	5,616 (11.1)	0.0008^*^
Rehabilitation (%)	189,849 (90.4)	51,667 (91.5)	40,279 (89.2)	52,348 (90.2)	45,555 (90.6)	<0.0001^*^
Admission to training and educational institution (%)	175,549 (83.6)	47,244 (83.7)	37,829 (83.8)	49,873 (85.9)	40,603 (80.7)	<0.0001^*^
Bed count	451 (326, 613)	424 (279, 615)	581 (344, 738)	419 (322, 547)	500 (350, 610)	<0.0001^†^
In-hospital mortality (%)	9,840 (4.68)	2,276 (4.03)	2,041 (4.52)	2,811 (4.85)	2,712 (5.40)	<0.0001^*^
**Rural**						
ACDI	1.90-0.52	1.90-1.24	1.24-1.06	1.06-0.85	0.85-0.52	―
Number of patients	266,296	66,828	66,323	66,542	66,603	―
JCS score upon admission						
0 (%)	128,616 (48.3)	30,173 (45.1)	30,304 (45.7)	33,015 (49.6)	35,124 (52.7)	<0.0001^*^
1-digit code (%)	100,583 (37.7)	27,587 (41.2)	26,689 (40.2)	24,107 (36.2)	22,200 (33.3)	<0.0001^*^
2-digit code (%)	24,510 (9.2)	6,015 (9.0)	6,234 (9.4)	6,281 (9.4)	5,980 (8.9)	0.4808
3-digit code (%)	12,516 (4.7)	3,051 (4.5)	3,069 (4.6)	3,135 (4.7)	3,261 (4.9)	0.0017
EMS utilization (%)	153,868 (57.7)	38,327 (57.3)	38,390 (57.8)	38,483 (57.8)	38,668 (58.0)	0.0078^*^
Reperfusion therapy (%)	26,295 (9.8)	6,822 (10.29)	6,635 (10.0)	6,664 (10.0)	6,174 (9.2)	<0.0001^*^
Rehabilitation (%)	239,508 (89.9)	60,309 (90.2)	59,958 (90.4)	59,777 (89.8)	59,464 (89.2)	0.2221^*^
Admission to training and educational institution (%)	222,147 (83.4)	58,192 (87.0)	57,663 (86.9)	54,276 (81.5)	52,016 (78.1)	<0.0001^*^
Bed count	482 (340, 609)	514 (400, 637)	440 (320, 605)	450 (325, 592)	500 (350, 661)	<0.0001^†^
In-hospital mortality (%)	14,968 (5.62)	3,398 (5.08)	3,653 (5.51)	3,709 (5.57)	4,208 (6.32)	<0.0001^*^
**Depopulated**						
ACDI	1.93-0.18	1.93-1.25	1.25-0.95	0.95-0.82	0.82-0.18	―
Number of patients	49,550	11,549	13,010	11,794	13,197	―
JCS score upon admission						
0 (%)	22,966 (46.3)	5,321 (46.0)	5,932 (45.6)	5,878 (49.8)	5,835 (44.2)	0.1617
1-digit code (%)	19,498 (39.3)	4,720 (40.8)	5,058 (38.8)	4,162 (35.2)	5,558 (42.1)	0.2550
2-digit code (%)	4,598 (9.2)	986 (8.5)	1,325 (10.1)	1,122 (9.5)	1,165 (8.8)	0.4599
3-digit code (%)	2,482 (5.0)	522 (4.5)	693 (5.3)	632 (5.3)	635 (4.8)	0.2039
EMS utilization (%)	26,878 (54.2)	5,833 (50.59)	6,827 (52.4)	6,506 (55.1)	7,712 (58.4)	<0.0001^*^
Reperfusion therapy (%)	3,902 (7.8)	837 (7.2)	945 (7.2)	966 (8.1)	1,154 (8.7)	<0.0001^*^
Rehabilitation (%)	43,425 (87.6)	9,748 (84.4)	11,077 (85.1)	10,675 (90.5)	11,925 (90.3)	<0.0001^*^
Admission to training and educational institution (%)	37,983 (76.6)	9,077 (78.6)	10,436 (80.2)	9,899 (83.9)	8,571 (64.9)	<0.0001^*^
Bed count	388 (300, 460)	410 (354, 514)	355 (304, 445)	354 (270, 490)	350 (271, 438)	<0.0001^†^
In-hospital mortality (%)	3,329 (6.71)	838 (7.26)	879 (6.76)	716 (6.07)	896 (6.79)	0.0272^*^

Bed count and hospitalization days are presented as medians (first and third quartiles). Other items are presented as frequencies (%). ^*^Cochran-Armitage trend test (one-sided); ^†^Jonckheere-Terpstra trend test (one-sided). ACDI, acute care density index; EMS, emergency medical services; JCS, Japan Coma Scale.

The trends based on home-to-hospital distance are presented in Table 3. As the distance increased in urban areas, there was an increase in the proportion of severe cases (JCS 3-digit code), along with an increase in EMS utilization, reperfusion therapy, and admission rates to accredited hospitals; in-hospital mortality also increased. Similar trends were observed in rural areas. Conversely, in depopulated areas, as the distance increased, there was an increase in the utilization of EMS, reperfusion therapy, rehabilitation, and admission rates to accredited hospitals; accordingly, a decrease in in-hospital mortality was observed.

**Table 3.** Trends in Severity, Care, and Outcomes According to the Home-to-Hospital Distance.

	Home-to-hospital distance					
	Overall	1st quartile	2nd quartile	3rd quartile	4th quartile	Trend test
						p-value
**Urban**						
Distance (km)	0-49.95	0-1.66	1.66-2.95	2.95-5.00	5.00-49.95	―
Number of patients	209,843	52,458	52,459	52,465	52,461	―
JCS score upon admission						
0 (%)	101,296 (48.2)	26,297 (50.1)	25,302 (48.2)	24,615 (46.9)	25,082 (47.8)	<0.0001^*^
1-digit code (%)	81,166 (38.6)	19,940 (38.0)	20,331 (38.7)	20,738 (39.5)	20,157 (38.4)	0.0175^*^
2-digit code (%)	18,572 (8.8)	4,307 (8.2)	4,668 (8.9)	4,810 (9.1)	4,787 (9.1)	<0.0001^*^
3-digit code (%)	8,767 (4.1)	1,906 (3.6)	2,142 (4.0)	2,293 (4.3)	2,426 (4.6)	<0.0001^*^
EMS utilization (%)	128,701 (61.3)	29,577 (56.3)	32,002 (61.0)	33,135 (63.1)	33,987 (64.7)	<0.0001^*^
Reperfusion therapy (%)	22,565 (10.7)	4,766 (9.0)	5,407 (10.3)	5,791 (11.0)	6,601 (12.5)	<0.0001^*^
Rehabilitation (%)	189,849 (90.4)	47,747 (91.0)	47,674 (90.8)	47,628 (90.7)	46,800 (89.2)	<0.0001^*^
Admission to training and educational institution (%)	175,549 (83.6)	42,104 (80.2)	43,459 (82.8)	44,753 (85.3)	45,233 (86.2)	<0.0001^*^
Bed count	451 (326, 613)	417 (317, 584)	440 (325, 610)	469 (329, 613)	499 (333, 659)	<0.0001^†^
In-hospital mortality (%)	9,840 (4.68)	2,400 (4.5)	2,435 (4.6)	2,491 (4.7)	2,514 (4.7)	0.0333^*^
**Rural**						
Distance (km)	0-49.99	0-2.63	2.63-5.14	5.14-9.62	9.62-49.99	―
Number of patients	266,296	66,570	66,562	66,588	66,576	―
JCS score upon admission						
0 (%)	128,616 (48.3)	33,308 (50.0)	33,047 (49.6)	32,322 (48.5)	29,939 (44.9)	<0.0001^*^
1-digit code (%)	100,583 (37.7)	24,808 (37.2)	24,584 (36.9)	24,763 (37.2)	26,428 (39.7)	<0.0001^*^
2-digit code (%)	24,510 (9.2)	5,591 (8.4)	5,910 (8.8)	6,259 (9.4)	6,750 (10.1)	<0.0001^*^
3-digit code (%)	12,516 (4.7)	2,841 (4.2)	3,008 (4.5)	3,232 (4.8)	3,435 (5.1)	<0.0001^*^
EMS utilization (%)	153,868 (57.7)	34,905 (52.4)	37,122 (55.7)	38,979 (58.5)	42,862 (64.3)	<0.0001^*^
Reperfusion therapy (%)	26,295 (9.8)	5,867 (8.8)	6,146 (9.2)	6,650 (9.9)	7,632 (11.4)	<0.0001^*^
Rehabilitation (%)	239,508 (89.9)	59,837 (89.8)	59,973 (90.1)	59,918 (89.9)	59,780 (89.7)	0.2221^*^
Admission to training and educational institution (%)	222,147 (83.4)	53,608 (80.5)	54,707 (82.1)	56,166 (84.3)	57,666 (86.6)	<0.0001^*^
Bed count	482 (340, 609)	470 (325, 595)	480 (343, 606)	489 (340, 630)	500 (350, 651)	<0.0001^†^
In-hospital mortality (%)	14,968 (5.62)	3,573 (5.3)	3,672 (5.5)	3,831 (5.7)	3,892 (5.8)	<0.0001^*^
**Depopulated**						
Distance (km)	0-49.97	0-3.64	3.64-8.71	8.71-17.06	17.06-49.97	―
Number of patients	49,550	12,387	12,387	12,377	12,399	―
JCS score upon admission						
0 (%)	22,966 (46.3)	6,169 (49.8)	5,852 (47.2)	5,723 (46.2)	5,222 (42.1)	<0.0001^*^
1-digit code (%)	19,498 (39.3)	4,601 (37.1)	4,896 (39.5)	4,870 (39.3)	5,131 (41.3)	<0.0001^*^
2-digit code (%)	4,598 (9.2)	1,016 (8.2)	1,062 (8.5)	1,113 (8.9)	1,407 (11.3)	<0.0001^*^
3-digit code (%)	2,482 (5.0)	600 (4.8)	574 (4.6)	670 (5.4)	638 (5.1)	0.0273^*^
EMS utilization (%)	26,878 (54.2)	5,613 (45.3)	6,171 (49.8)	6,883 (55.6)	8,211 (66.2)	<0.0001^*^
Reperfusion therapy (%)	3,902 (7.8)	709 (5.7)	812 (6.5)	901 (7.2)	1,480 (11.9)	<0.0001^*^
Rehabilitation (%)	43,425 (87.6)	10,695 (86.3)	10,833 (87.4)	10,915 (88.1)	10,982 (88.5)	<0.0001^*^
Admission to training and educational institution (%)	37,983 (76.6)	8,853 (71.4)	9,639 (77.8)	9,436 (76.2)	10,055 (81.1)	<0.0001^*^
Bed count	388 (300, 460)	354 (300, 426)	354 (300, 435)	379 (304, 456)	435 (320, 558)	<0.0001^†^
In-hospital mortality (%)	3,329 (6.71)	881 (7.1)	848 (6.8)	850 (6.8)	750 (6.0)	0.0008^*^

Bed count and hospitalization days are presented as medians (first and third quartiles). All other items are presented as frequencies (%). ^*^Cochran-Armitage trend test (one-sided); ^†^Jonckheere-Terpstra trend test (one-sided). EMS, emergency medical services; JCS, Japan Coma Scale.

### Association between ACDI and home-to-hospital distance and in-hospital mortality

The results of the generalized linear mixed models assessing the association between ACDI and in-hospital mortality are presented in Table 4. In both urban and rural areas, an association was observed between lower ACDI and in-hospital mortality (adjusted odds ratio for urban: Q2 = 1.16 [1.02-1.31], Q3 = 1.23 [1.10-1.39], Q4 = 1.35 [1.19-1.53]; rural: Q2 = 1.12 [0.99-1.26], Q3 = 1.15 [1.02-1.29], Q4 = 1.25 [1.11-1.42]); however, no such association was observed in depopulated areas. The association between home-to-hospital distance and in-hospital mortality is presented in Table 4. In depopulated areas, there was an association indicating lower in-hospital mortality when home-to-hospital distance exceeded the median (Q3 = 0.84 [0.74-0.95], Q4 = 0.78 [0.68-0.89]); conversely, no such associations were observed in urban and rural areas.

**Table 4.** Association between ACDI and Home-to-Hospital Distance and in-Hospital Mortality.

	Model 1			Model 2			Model 3		
	OR	95% CI	p-value	AOR	95% CI	p-value	AOR	95% CI	p-value
**Association between ACDI and in-hospital mortality**									
**Urban**									
First quantile	Ref.			Ref.			Ref.		
Second quantile	1.130	1.018-1.254	0.0223	1.160	1.046-1.288	0.0051	1.161	1.022-1.318	0.0216
Third quantile	1.103	0.998-1.219	0.0560	1.199	1.086-1.324	0.0003	1.238	1.100-1.393	0.0004
Fourth quantile	1.126	1.009-1.256	0.0332	1.277	1.147-1.421	<0.0001	1.354	1.193-1.538	<0.0001
**Rural**									
First quantile	Ref.			Ref.			Ref.		
Second quantile	1.062	0.958-1.178	0.2548	1.081	0.978-1.196	0.1260	1.120	0.992-1.264	0.0671
Third quantile	1.120	1.016-1.235	0.0223	1.156	1.052-1.271	0.0027	1.150	1.025-1.290	0.0172
Fourth quantile	1.174	1.058-1.303	0.0026	1.293	1.169-1.430	<0.0001	1.259	1.112-1.425	0.0003
**Depopulated**									
First quantile	Ref.			Ref.			Ref.		
Second quantile	0.843	0.695-1.023	0.0844	0.874	0.725-1.054	0.1600	0.895	0.732-1.093	0.2761
Third quantile	0.806	0.666-0.976	0.0276	0.832	0.691-1.002	0.0520	0.952	0.779-1.164	0.6315
Fourth quantile	0.899	0.750-1.077	0.2483	0.980	0.822-1.169	0.8237	1.053	0.871-1.274	0.5925
**Association between home-to-hospital distance and in-hospital mortality**									
**Urban**									
First quantile	Ref.			Ref.			Ref.		
Second quantile	1.010	0.954-1.073	0.689	1.018	0.960-1.080	0.5511	0.961	0.894-1.032	0.2747
Third quantile	1.040	0.981-1.103	0.188	1.066	1.005-1.131	0.0350	0.961	0.894-1.033	0.2822
Fourth quantile	1.080	1.020-1.150	0.008	1.187	1.118-1.261	<0.0001	0.964	0.895-1.038	0.3299
**Rural**									
First quantile	Ref.			Ref.			Ref.		
Second quantile	1.043	0.993-1.094	0.0909	1.054	1.003-1.107	0.0358	0.999	0.942-1.060	0.9745
Third quantile	1.085	1.034-1.139	0.0010	1.101	1.048-1.157	0.0001	0.993	0.935-1.054	0.8145
Fourth quantile	1.120	1.066-1.178	<0.0001	1.158	1.101-1.217	<0.0001	0.959	0.902-1.020	0.1856
**Depopulated**									
First quantile	Ref.			Ref.			Ref.		
Second quantile	0.980	0.886-1.084	0.6961	0.944	0.853-1.046	0.2700	0.903	0.799-1.021	0.1027
Third quantile	0.980	0.885-1.085	0.7004	0.935	0.844-1.037	0.2034	0.842	0.743-0.953	0.0064
Fourth quantile	0.909	0.812-1.017	0.0949	0.927	0.828-1.037	0.1860	0.782	0.681-0.898	0.0005

Model 1: Unadjusted regression model for per capita acute care density (in four quartile categories) and in-hospital mortality. Model 2: Model adjusted for age and sex relative to Model 1. Model 3: Model adjusted for per capita acute care density (in quartile categories), distance from residence to hospital (in quartile categories), age, sex, hypertension, hyperlipidemia, diabetes mellitus, congestive heart failure, atrial fibrillation, renal disease, obesity, smoking, pre-stroke mRS score (6 categories, 0-5), admission JCS (4 categories, 0-3), EMS utilization, implementation of reperfusion therapy, implementation of brain rehabilitation, admission to a training and educational institution, and bed count in the admitting hospital as covariates with respect to Model 1. ACDI, acute care density index; OR, odds ratio; CI, confidence interval; AOR, adjusted odds ratio; Ref, reference; mRS, modified Rankin scale; JCS, Japan Coma Scale; EMS, emergency medical services.

## Discussion

We assessed the association between ACDI and home-to-hospital distance and in-hospital mortality in patients with AIS stratified into three groups (urban, rural, and depopulated) based on the population size of their residence area. The main conclusions were as follows: a lower ACDI was associated with higher in-hospital mortality in both urban and rural areas. However, longer home-to-hospital distance was not necessarily associated with higher in-hospital mortality.

Regarding the nationwide distribution of ACDI, blue (quite low ACDI) was scattered across the countryside, indicating an absolute shortage of medical resources owing to geographic barriers, low population density, and insufficient infrastructure. Light blue (low ACDI) was concentrated in metropolitan areas, reflecting a relative shortage driven by high population density and medical demand. Red and pink (sufficient ACDI) in the countryside suggest that some regions have surplus resources relative to population size, thereby implicating the need for redistribution based on population and geography (Figure 2A). Considering home-to-hospital distances, blue and green (long distances) were mainly observed in low population areas, particularly in parts of Hokkaido, Aomori, and Shiga, with median distances exceeding 40 km; these long distances reflected dispersed hospitals and natural barriers, implying the need for better emergency transport systems and telemedicine. Meanwhile, red (short distances) in metropolitan areas such as Tokyo and Osaka highlighted closer proximity to hospitals. However, these areas face challenges such as overcrowding and delayed care owing to high population density, emphasizing the need for improved triage systems and more efficient resource allocation. In lowly populated areas, red may indicate that hospitals are located in narrow habitable regions such as basins, where residences are not widely distributed. (Figure 2B).

In urban and rural areas, reperfusion therapy decreased in areas with lower ACDI, whereas it increased in depopulated areas; these paradoxical results may indicate the features of emergency systems in depopulated areas. Regions with fewer medical resources may have robust planned systems to support emergency patients, such as doctor helicopters or drip-and-ships, and patients may be transported more rapidly to hospitals that can provide tissue plasminogen activator and endovascular thrombectomy. According to a Nara Doctor Helicopter report, several dispatches take place in mountainous regions with scarce medical resources ^(30)^.

The proportion of patients who received reperfusion therapy tended to increase with increasing home-to-hospital distance, regardless of secondary medical area categories; this finding contradicted our initial hypothesis that longer home-to-hospital distances would lead to prolonged transport times and a lower proportion of patients receiving reperfusion therapy within a critical time window. One explanation could be the use of doctor helicopters for long-distance transport ^(31)^. Patients transported by helicopter receive prehospital care from specialized medical staff during the flight; this approach allows for the early determination of indications for reperfusion therapy and rapid transport to hospitals capable of performing the therapy. As of February 2024, 57 doctor helicopters have been deployed across Japan’s 47 prefectures. This system plays a crucial role in overcoming geographical barriers to acute care ^(32)^.

Regarding in-hospital mortality, Model 2, which was adjusted for sex and age, showed an association between lower ACDI and higher in-hospital mortality in urban and rural areas. Furthermore, the association did not disappear in Model 3, which was adjusted for medical history, severity, care, and hospital factors. This result suggests that in urban and rural areas, ACDI of a patient’s residence may play an important role in influencing survival outcomes. Regarding home-to-hospital distance, in urban and rural areas, Model 2 was associated with higher in-hospital mortality as the distance increased; however, this association disappeared in Model 3, suggesting that modifiable factors, including EMS utilization, care, and hospital factors, could partially control mortality risk in areas far from hospitals. In contrast, in depopulated areas, in-hospital mortality decreased with increasing distance from the hospital. One possible reason could be the well-developed emergency systems in remote locations, such as doctor and fire department helicopters deployed in mountainous regions or wide-area emergency zones. Patients who live far from hospitals may be transported rapidly to acute care hospitals with advanced medical equipment and specialized stroke staff. Another possible explanation is that many patients residing far from the hospital were either not transported or died before admission; this could have skewed the data, resulting in people living farther away appearing to have better survival outcomes.

Studies evaluating the association between the number of stroke specialists per 100,000 people and the number of surgical procedures per year with patient outcomes ^(20), (33)^ are important for quantitatively assessing regional healthcare accessibility. Such indicators measure the demand-supply balance of medical services in blocks separated by administrative divisions, such as secondary medical areas and prefectures ^(34), (35), (36)^. However, in practice, patients are often transferred across secondary medical areas and prefectural boundaries ^(37), (38)^. This study evaluated the association between ACDI and home-to-hospital distance and in-hospital mortality that was not restricted to administrative boundaries; this may help us understand the association between acute care accessibility and in-hospital mortality, considering real patient mobility.

Existing studies on acute care accessibility and outcomes in patients with AIS have explored the disparities between urban and rural areas ^(39)^, the association between the geographic distribution of healthcare resources and quality of care ^(40)^, and the impact of travel time to hospitals on in-hospital mortality ^(41)^. In this study, we analyzed accessibility using ACDI and home-to-hospital distance across Japan and examined the association of these indicators with in-hospital mortality.

### Future direction

First, since the present study could not consider the impact of the number of stroke specialists, PSCs, and comprehensive stroke center certification hospitals in the region, further studies considering these factors will be conducted. Second, an AIS care density index that replaces the number of general anesthesia cases with the number of tissue plasminogen activator and endovascular thrombectomy cases will be developed; this index is more directly associated with the prognosis of AIS as a regional accessibility index to clarify its association with discharge outcomes. Third, patient outcomes considering the impact of prehospital care from disease onset to hospital arrival, as suggested by Aref et al. ^(42)^ and Kamal et al. ^(43)^, will analyze a patient registry that collects prehospitalization information.

### Study limitations

First, the results of this study should be interpreted with caution because it is a retrospective observational study in which only associations, not causalities, were assessed. Second, the cardiovascular database used in this study was sponsored by the Japanese Circulation Society and was not specific to stroke. However, based on the estimated number of 142,500 hospitalized patients with cerebrovascular diseases in 2017 ^(44)^, and if two-thirds to three-quarters of these patients had ischemic stroke ^(45)^, approximately 100,000 people were admitted to hospitals annually. The data we analyzed over a 6-year period, comprising 620,000 cases, might have covered most patients with ischemic stroke in Japan. Nevertheless, since information on treatments and outcomes at stroke-specialized hospitals was missing, the results should be interpreted with caution. Third, patient residence and AIS onset location might not be associated. Moreover, although excluding cases of onset outside the home was required, this was not possible owing to the lack of an identification flag in the available data. However, the median age of patients who developed AIS was ≥75 years, and most cases occurred during the night or when waking up; therefore, there is a high possibility that AIS occurred while the patient was at home. Fourth, prehospital factors were not fully detailed in this study. Although EMS utilization was included as a confounding factor, we could not distinguish between ground ambulance and air transportation. This limitation might have affected the interpretation of the results, as it did not account for the differences between air and ground transport in terms of speed and access to advanced care, potentially obscuring their specific impacts on mortality rates. Finally, important factors such as regional classification, demographics, severity, EMS utilization, reperfusion therapy, and the accreditation of training and teaching hospitals, as well as the number of beds, were considered confounding factors. However, laboratory tests, drug therapy, number of specialists, and stroke center accreditation were not considered. Previous studies have shown that these factors are associated with in-hospital mortality ^(25)^; their omission might have affected the validity of the findings on the association between acute care accessibility and mortality.

### Conclusion

A lower ACDI was associated with higher in-hospital mortality in both urban and rural areas. However, longer home-to-hospital distance was not necessarily associated with higher in-hospital mortality.

## Article Information

### Conflicts of Interest

None

### Sources of Funding

This work was supported by JSPS KAKENHI (grant number 20K10492) and The Health Care Science Institute Research Grant 2021.

### Acknowledgement

We thank Ms. Sumita, Dr. Nakai, Dr. Iwanaga, and Dr. Miyamoto from the JROAD office for their support in preparing and analyzing the data for the study.

### Author Contributions

Y. Sasahara, Y. Gon, and E. Hida designed the study, performed data acquisition, analyzed and interpreted data, and drafted the manuscript or revised it critically.

### Approval by Institutional Review Board (IRB)

This study was approved by the Research Ethics Review Board of the National Cerebral and Cardiovascular Center (R21041).

### Data Availability Statement

The data supporting the results of this study were used under a license from The Japanese Society of Cardiology and are not publicly available without permission.
